# Twik‐2^−/−^ mouse demonstrates pulmonary vascular heterogeneity in intracellular pathways for vasocontractility

**DOI:** 10.14814/phy2.13950

**Published:** 2019-01-10

**Authors:** Melanie G. Kitagawa, Julia O. Reynolds, David Durgan, George Rodney, Harry Karmouty‐Quintana, Robert Bryan, Lavannya M. Pandit

**Affiliations:** ^1^ Baylor College of Medicine Houston Texas; ^2^ Texas Children’s Hospital Houston Texas; ^3^ University of Texas‐Houston McGovern Medical School Houston Texas; ^4^ Michael E.DeBakey Veterans Affairs Medical Center Houston Texas

**Keywords:** Potassium channels, pulmonary artery smooth muscle cells, pulmonary hypertension, vasocontractility

## Abstract

We have previously shown Twik‐2^−/−^ mice develop pulmonary hypertension and vascular remodeling. We hypothesized that distal pulmonary arteries (D‐PAs) of the Twik‐2^−/−^ mice are hypercontractile under physiological venous conditions due to altered electrophysiologic properties between the conduit and resistance vessels in the pulmonary vascular bed. We measured resting membrane potential and intracellular calcium through Fura‐2 in freshly digested pulmonary artery smooth muscles (PASMCs) from both the right main (RM‐PA) and D‐PA (distal) regions of pulmonary artery from WT and Twik‐2^−/−^ mice. Whole segments of RM‐PAs and D‐PAs from 20 to 24‐week‐old wildtype (WT) and Twik‐2^−/−^ mice were also pressurized between two glass micropipettes and bathed in buffer with either arterial or venous conditions. Abluminally‐applied phenylephrine (PE) and U46619 were added to the buffer at log increments and vessel diameter was measured. All values were expressed as averages with ±SEM. Vasoconstrictor responses did not differ between WT and Twik‐2^−/−^ RM‐PAs under arterial conditions. Under venous conditions, Twik‐2^−/−^ RM‐PAs showed an increased sensitivity to PE with a lower EC50 (*P* = 0.02). Under venous conditions, Twik‐2^−/−^ D‐PAs showed an increase maximal vasoconstrictor response to both phenylephrine and U46619 compared to the WT mice (*P* < 0.05). Isolated PASMCs from Twik‐2 ^−/−^ D‐PA were depolarized and had higher intracellular calcium levels compared to PASMCs from RM‐PA of both WT and Twik‐2^−/−^ mice. These studies suggest that hypercontractile responses and electrophysiologic properties unique to the anatomic location of the D‐PAs may contribute to pulmonary hypertensive vasculopathy.

## Introduction

Pulmonary hypertension is a complex disease resulting in a progressive increase in pulmonary vascular resistance leading to right‐sided heart failure and often, death. The vascular remodeling that is pathognomic for pulmonary hypertension is characterized by a distal resistance vessel arteriopathy affecting the smaller diameter resistance vessels in the pulmonary vascular bed. This resistance vessel arteriopathy occurs as a result of many proposed molecular mechanisms that contribute to endothelial cell hyperplasia and smooth‐muscle cell hypertrophy leading to decreased luminal diameter and increased pulmonary vascular resistance (Schermuly et al. 2011; Sutendra and Michelakis 2013; Galiè et al. 2015; Ryan and Archer 2015).

Recently, a form of hereditary pulmonary hypertension due to heterozygous loss of function mutations in the KCNK3 gene encoding the potassium ion channel, Task‐1, was identified (Lloyd et al. 2011; Bohnen et al. 2017). Task‐1 is an acid‐sensitive potassium channel, which contributes to the resting potential of human pulmonary artery smooth muscle cells and is a member of the six subfamilies included in the K_2_P two‐pore potassium channel family that were first described in 1996 and are found in the cerebral, peripheral, and pulmonary vascular system (Salinas et al. 1999; Patel et al. 2000; Gardener et al. 2004; Olschewski et al. 2006; Enyedi and Czirjak 2010; Lloyd et al. 2011; Ma et al. 2013; Antigny et al. 2016). Our previous work demonstrated that loss‐of‐function of another K_2_P channel, Twik‐2, led to the development spontaneous elevation of right ventricular systolic pressures and microvascular arteriopathy consistent with a phenotype of pulmonary hypertension (Pandit et al. 2014). Twik‐2^−/−^ mice pulmonary vascular rings demonstrated hypercontractility and in this study, we demonstrate that this vascular hypercontractility is specifically targeted to the smaller resistance vessels of the distal vasculature, the pathologic site of vessel injury. As the defining vasculopathy of pulmonary hypertension is known to preferentially target the resistance vessels of the distal pulmonary vasculature, we hypothesized that differences in membrane potential and vasoreactivity between the proximal conduit vessels and the resistance vessels in the distal locations of the Twik‐2^−/−^ mouse pulmonary vasculature contribute to the site‐specific microvasculopathy of pulmonary hypertension. These studies not only demonstrate a potential mechanism behind the Twik‐2 (and K_2_P) related hypercontractility, but also importantly reveal that functional characteristics of the distal vasculature contribute to selective distal vessel hypercontractility. Moreover, we predicted that alteration of the environmental conditions of the vasoreactivity experiments to reflect the venous physiology of the pulmonary vascular milieu would enhance the accuracy of vasoreactivity in the pulmonary vessels. As previous vascular physiologic studies in animal models have focused techniques on the proximal vasculature and arterial physiologic conditions, our current findings are a significant alteration in the way we study the pathophysiology of pulmonary hypertension, focusing mechanistic studies to the site of pathology in the distal vasculature and under accurate venous conditions.

## Methods

### Animals

All studies were approved by the Institutional Animal Care and Use Committee (IACUC) of Baylor College of Medicine. Mice used in these studies were male mice at 20 to 24 weeks of age. Twik‐2^−/−^ and wildtype littermate mice on a SV129xC57BL/6 background were used for all experiments (Pandit et al. 2014).

### Isolation of pulmonary artery vessels

Mice were anesthetized with isoflurane and the lungs were rapidly removed en‐block and placed in cold Krebs buffer. Either the right main pulmonary artery (RM‐PA), measuring approximately 1 mm in diameter, or the distal second‐order pulmonary right artery (D‐PA), measuring approximately 500 microns in diameter, was carefully harvested and surrounding tissue was removed, ensuring the section of vessel had no side branches or other holes.

### Isolating pulmonary artery smooth cells (PASMCs) through enzymatic digestion:

After surgical isolation of either the right main or distal second‐order branch of the pulmonary artery from Twik‐2^−/−^ and WT littermates, vessels were minced and treated with a papain/dithiothreitol (Sigma) cocktail (15 min at 37°C), followed by a collagenase/hyaluronidase (Sigma) cocktail (30 min at 37°C). The mixture was then triturated to form a suspension of dispersed PASMCs which was placed on ice and typically used between 2 and 6 h after isolation for electrophysiology studies or seeded in culture for calcium measurements.

### Patch‐clamping and measurement of membrane potential in pulmonary artery smooth muscle cells (PASMCs)

A mixture of freshly dispersed PASMCs obtained by enzymatic dissociation were plated on a coverslip (10–40 cells/coverslip). Using an Axopatch 200B integrated patch clamp amplifier and pClamp version 9.2 software, membrane potent (Vm) using current clamp was measured in the whole‐cell model. Current density using voltage clamp was also measured and compared between PASMCs from Twik‐2^−/−^ and WT littermates. Circulating buffer isoelectric and molecular concentrations are as previously described in our published work (Lloyd et al. 2011).

### Measurement of intracellular calcium in pulmonary artery smooth muscle cells (PASCMs)

Freshly isolated PASMCs were seeded and allowed to adhere to a glass coverslip placed in 35 mm dish and cultured with M199 media+10% fetal bovine serum and 1%Pen/Strep until 50% confluence (about 3 days). One coverslip was fixed and incubated with mouse smooth muscle α‐actin antibody (Immunoresearch #015‐000‐003) followed by incubation with Alexa Fluor 594 goat anti‐mouse IgG (Molecular Probes‐A11005) as previously described in order to confirm identity of the cultured PASMCs. (Pandit et al. 2014) Next, cells were incubated in the dark with fura‐2 AM, (2.5 *μ*mol/L, Teflabs, TX, USA) plus Pluronic F‐127 (0.01%) for 1 h, and then washed for 15 min with physiologic saline solution (PSS) of the following composition (in mmol/L): 137 NaCl, 5.6 KCl, 1.6 CaCl_2_, 1 MgCl_2_, 10 glucose, and 10 HEPES, with pH adjusted to 7.4 with NaOH. A Sutter Lamda DG‐5 Ultra high‐speed wavelength changer was used to excite Fura‐2 at 340/10 nm and 380/10 nm and the emission intensity was collected at 510/80 nm on a charge coupled device (CCD) Camera (CoolSNAP MYO, Photometrics, Tucson, AZ) attached to an Axio Observer (Zeiss) inverted microscope (20× objective, 0.5 NA) and images were captured every 3 sec. Values were reported as separate values at 340 and 380 nm and the 340/380 excitation ratio was calculated for each cell, with increased ratio reflective of increased intracellular Ca^2+.^ Measuring by ratio considerably reduces the potentially confounding effects of variations in cell thickness, dye uptake, and photobleaching.

### Pressurizing pulmonary vessels

After surgical extraction, the vessel was immediately placed into the vessel chamber containing Krebs buffer consisting of (mmol/L): 119 NaCl, 4.7 KCl, 1 MgSO_4_, 1.2 KH_2_PO_4_, 0.026 EDTA, 1.6 CaCl_2_, 5.5 glucose, and 25 NaHCO_3_. The buffer was gassed with oxygen and carbon dioxide to create two different conditions, one similar to systemic arterial blood (higher oxygen tension) or one similar to venous blood (lower oxygen tension). The pulmonary artery physiologic conditions are venous and a venous buffer would more closely mimic these lower oxygen tension conditions even though previous pressurized vessel studies have conventionally utilized buffer with higher oxygen tension. The standard “arterial” buffer was created using 20% oxygen and 5% CO_2_ to create a pH of 7.39 (SD 0.02) and a pO_2_ of 165 mmHg (SD 14 mmHg). The venous (lower oxygen tension) buffer was created using 0% oxygen and 5% CO_2_ to create a pH of 7.31 (SD 0.03) and a pO_2_ of 45 mmHg (SD 11 mmHg). The open ends of the vessel were mounted on glass micropipettes of an appropriate diameter for the size of the opening of the vessel and secured to the pipettes with 12–0 nylon suture. The internal lumen of the vessel was not exposed to air throughout the preparation to ensure the integrity of the endothelium. The vessel was pressurized with a column of buffer connected to the micropipettes and the vessel was tested for leaks ensuring the vessel maintained pressure in a closed system. The vessels were warmed to 37°C and held at a pressure of 5 mmHg for 30 min to allow equilibration. The vessels were magnified and digitally recorded.

### Tone development

In preparation to measuring the vessel diameter response to the addition of vasoconstrictors, we first assessed for the development of spontaneous tone following equilibration of RM‐PA and D‐PA vessels. For the RM‐PA, the vessel's external diameter was measured at 5 mmHg and then again at incrementally increased pressures of 15 mmHg and 30 mmHg. For the D‐PA, the vessel's external diameter was measured at 5 mmHg, then again at incrementally increased pressures of 10 mmHg and 15 mmHg. Once tone development assessments were completed, then pressure was reduced back to 15 mmHg for the RM‐PA and 10 mmHg for the D‐PA, and the vessel was allowed to equilibrate at this pressure of 10 mmHg for the completion of the experiments and addition of vasoconstrictor.

### Vasoconstrictor responses

Differences in vessel diameter to vasoconstrictors were calculated from a starting baseline diameter measured at physiologic representative pressures. These baseline pressures were based on our previous invasive hemodynamic measurements in these mice and our previous vasoreactivity studies in cerebral vessels of similar size and caliber. The RM‐PA vessels were maintained at a pressure of 15 mmHg, and D‐PA were maintained at 10 mmHg. Vasoconstrictor dose responses were measured in pressurized vessels using phenylephrine (PE) and U46619, a thromboxane A_2_ agonist that is known to activate Rho‐kinase. Vasoconstrictor was added to the Krebs buffer abluminally at increasing concentrations from 10^−10^mol/L to 10^−5^mol/L. At each concentration, the vessel was allowed to equilibrate in the vasoconstrictor solution for 15 minutes and the vessel external diameter was measured at the end of the equilibration.

### Data analysis and statistics

The images collected throughout the experiments were analyzed offline using edge detection software to measure the external diameter of the vessels. Percent constriction was calculated by: % constriction = 100 x (D_Tone_ – D_Resp_)/D_Tone_ where D_Tone_ is the vessel diameter at equilibrium prior to the exposure to vasoconstrictor and D_Resp_ is the vessel diameter at equilibrium following exposure to phenylephrine. Data are expressed as mean ± SEM. Dose response analysis was done using a two‐way repeated measure ANOVA. Analysis of EC_50_ values were calculated using one‐site specific binding nonlinear curve fitting with Prism 7 (GraphPad Software, Inc. San Diego, CA) for each vessel. EC_50_ values between two groups were compared using a Student's t‐test. Differences were consider statistically significant if *P* < 0.05.

## Results

### Twik‐2^−/−^ pulmonary artery smooth muscle cells (PASMCs) display heterogeneity in resting membrane potential (V_m_) based on anatomic location

**Figure 1:**
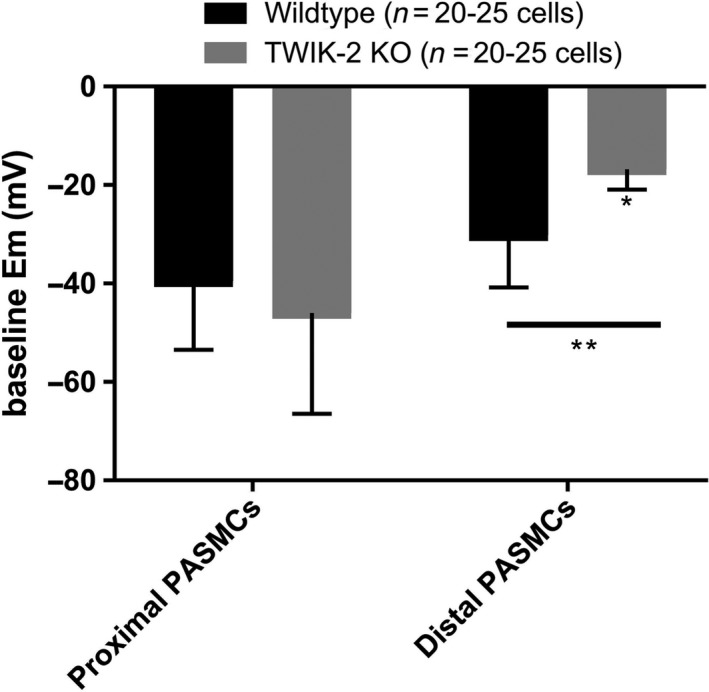
Resting membrane potential of freshly dispersed PASMCs. PASMCs digested and dispersed from right main (RM‐PA; proximal) vessel location and intralobar (D‐PA; distal) vessel location from both wildtype and Twik‐2^−/−^ mice. (P* < 0.05 between distal Twik‐2^−/−^ and distal WT cells; P** < 0.01 between proximal and distal PASMCs.) *n* = 5 animals per genotype.

We compared the V_m_ of PASMCs from Twik‐2^−/−^ and WT mice isolated from the RM‐PA and distal intralobar branch (D‐PA). (Fig. 1) While there were no significant differences in the Vm between WT and Twik‐2^−/−^ vascular smooth muscle cells from the proximal RM‐PA, the Twik‐2^−/−^ distal vascular smooth muscle cells were significantly more depolarized compared to WT distal vascular smooth cells (*P < 0.05) and there was an overall anatomic difference between the proximally derived cells versus those from the more distal anatomic region of the vasculature (**P < 0.01).

### Twik‐2^−/−^ pulmonary artery smooth muscle cells (PASMCS) demonstrated increased intracellular calcium levels compared to WT PASMCS

**Figure 2:**
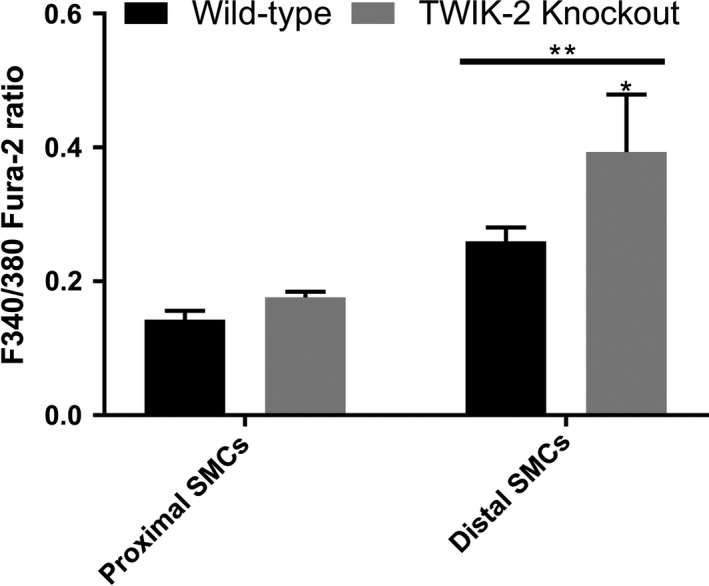
Baseline F340/380 ratio of cultured PASMCs from right main (proximal) vessel location and intralobar (distal) vessel location from both wildtype and TWIK‐2 ^−/−^ mice. (P** < 0.01 between all distal and proximal cells, P* < 0.05 between distal WT and distal Twik‐2^−/−^ PASMCs.) *n* = 5 animals per genotype, 12–15 cells per animal, per location. Overall anatomic variation between {Ca^2+^} approached statistical significance (*P* = 0.06).

Using Fura‐2, we measured intracellular calcium levels in PASMCs isolated from Twik‐2^−/−^ and WT RM‐PA and a distal intralobar branch (Fig. 2A). Distal intralobar PASMCs from Twik‐2^−/−^ mice demonstrated an increased resting (i.e. basal) fura‐2 fluorescence ratio compared to WT distal PASMCs and were overall higher than both WT and Twik‐2 PASMCs derived from the proximal region of the vasculature (Fig. 2B), suggesting an increase in resting [Ca^2+^]_i_. Similar to our observations in the electrical properties of these PASMCs (Fig. 1), the anatomic and genotype origin of the pulmonary vascular cells affected baseline intracellular Ca^2+^. Twik‐2^−/−^ distal intralobar PASMCs demonstrated higher levels of intracellular calcium and a more depolarized resting Vm compared to proximally derived PASMCs and WT PASMCs. The ratio in distal intralobar PASMCs was increased in the knockout mice compared to the wildtype but this increase did not achieve statistical significance.

### Twik‐2^−/−^ mice right main pulmonary artery (RM‐PA) did not show altered vasoreactivity to phenylephrine compared to WT RM‐PA when exposed to systemic arterial conditions

**Figure 3:**
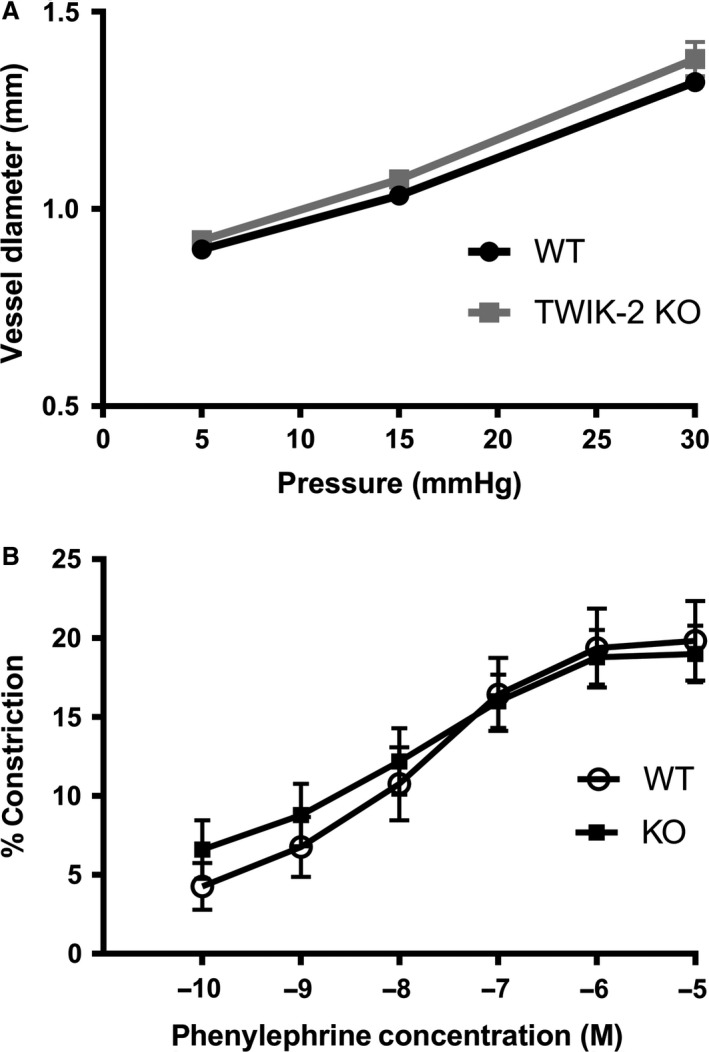
RM‐PA from Twik‐2^−/−^ and WT mice on different backgrounds in arterial (systemic)buffer. (A) Measurement of vessel diameter with increasing pressures evaluating for myogenic tone, which was not present. (B) Dose titration response curve to phenylephrine *n* = 5–6 animals per group.

With increasing amounts of pressure, the RM‐PA vessels continued to dilate without any decrease in diameter indicative of the absence myogenic tone in the main pulmonary vessels (Fig. 3A). Once equilibrated at 10 mmHg, and phenylephrine was added at increasing dose, the measured vessel diameters between WT and Twik‐2^−/−^ RM‐PAs did not demonstrate any differences in vasoreactivity in physiologic conditions of the postcapillary pulmonary vascular bed, which is the same as arterial/systemic oxygenated conditions (Fig. 3B).

### Twik‐2^−/−^ mice right main pulmonary artery (RM‐PA) showed increased vasoreactivity to phenylephrine when exposed to physiologic venous conditions of the pulmonary artery compared to WT RM‐PA exposed to the same physiologic conditions

**Figure 4:**
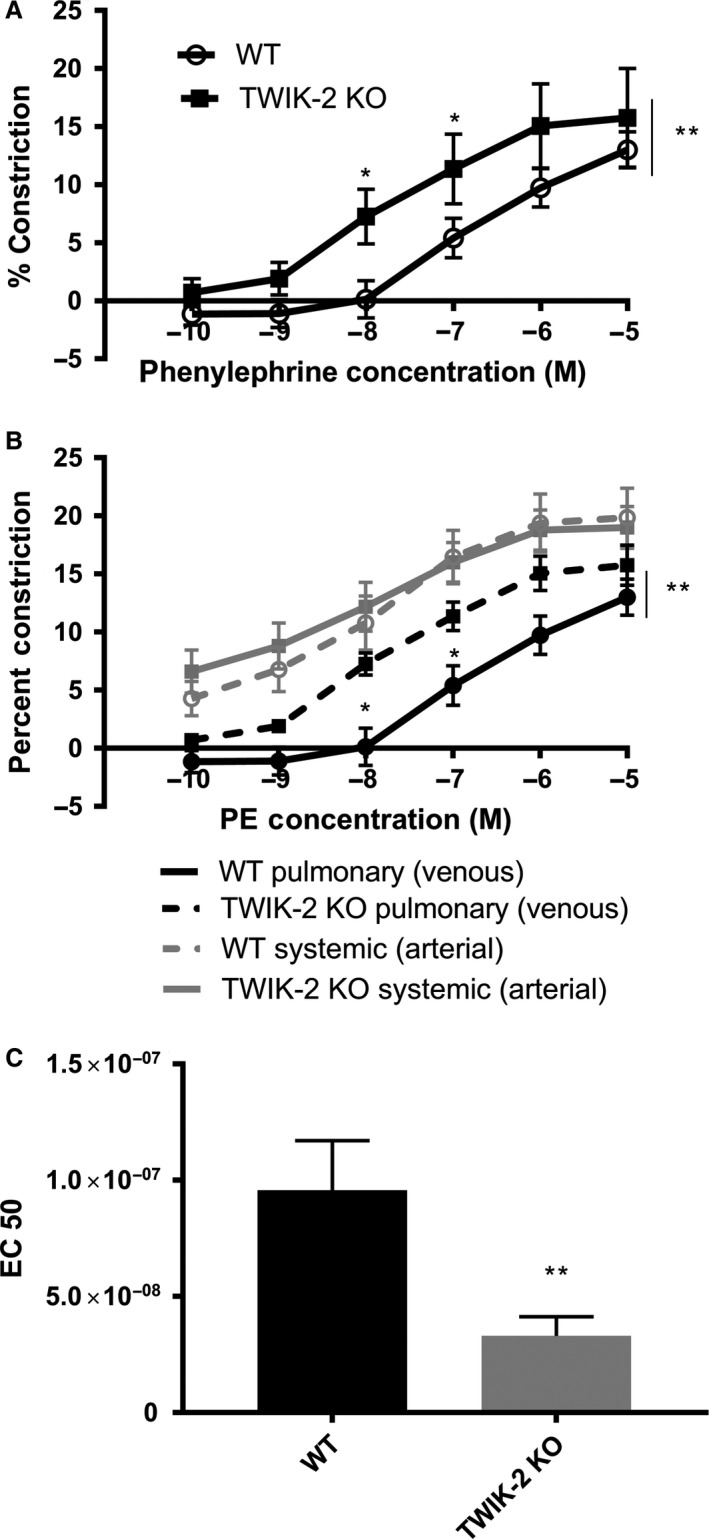
RM‐PA from Twik‐2^−/−^ and WT mice in PA(venous) conditions buffer. (A) Phenylephrine dose titration curve showing increased vasoreactivity in the TWIK‐2^−/−^. (B) Combined phenylephrine dose titration curve of the RM‐PA in venous and arterial conditions. (Fig 3A and 4A) (C) EC 50 for RM‐PA in PA conditions (venous.)

We then compared vasoreactivity in Twik‐2^−/−^ and WT RM‐PA vessels in physiologic conditions mimicking the pulmonary artery (venous or precapillary conditions) by adjusting pH and pO_2_ (mean pH 7.31 and mean pO_2_ 45 mmHg). By replicating the low oxygen tension milieu of the pulmonary vascular bed, differences in vasoreactivity between the Twik‐2^−/−^ and WT vessels that were not evident in systemic (arterial) conditions became apparent (Fig. 4A). Like the arterial conditions, resting tone did not develop in venous conditions (data not shown.) Figure 4B shows the combined data of vessel reactivity from the RM‐PA in both systemic and pulmonary arterial physiologic buffer conditions. After observing the variation in vasoreactivity of RM‐PA between systemic (arterial) and physiologic (venous) conditions, we determined the EC_50_ of the phenylephrine titration curves from RM‐PA vessels in physiologic buffer conditions. Figure 4C shows that Twik‐2^−/−^ RM‐PA vessels in venous conditions had a significantly lowed EC_50_ value compared to the WT values (*P* = 0.02), indicating an increased sensitivity to PE in the RM‐PA even though there was no change in vasocontractility at the highest doses.

### Distal pulmonary arteries (D‐PA) have altered vasoreactivity compared to the proximal right main pulmonary artery (RM‐PA) in physiologic venous conditions

**Figure 5:**
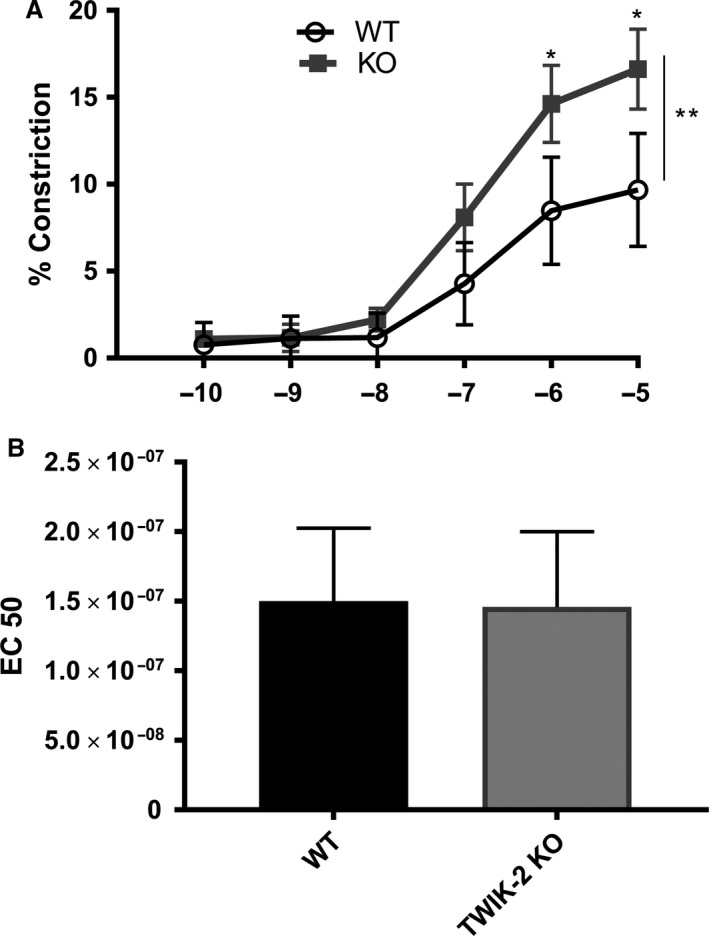
Distal‐PA >from Twik‐2^−/−^ and  WT mice in PA conditions (venous) buffer. (A) Phenylephrine dose titration curve showing altered vasoreactivity in the TWIK‐2 KO (*P < 0.05), particularly at the higher concentrations of phenylephrine, and overall effect (***P < 0.001). (B) EC50 for WT and Twik‐2^−/−^ D‐PA vessels in response to phenylephrine. *n* = 5–6 animals per group.

Figure 5A shows the phenylephrine titration curve of the D‐PA in physiologic (venous) buffer with heightened contractility in Twik‐2^−/−^ D‐PA compared to WT D‐PA to phenylephrine than was previously seen in the proximal vasculature. The Twik‐2^−/−^ D‐PA vessels appear slower to react to the PE but EC50 to phenylephrine between the WT and Twik‐2^−/−^ D‐PA were not significantly different (Fig. 5B). Similar to what was observed in proximal vessels, D‐PA vessels do not develop myogenic tone. (data not shown)

### Twik‐2^−/−^ D‐PA showed increased vasoreactivity to rho‐kinase activation with U46619

**Figure 6:**
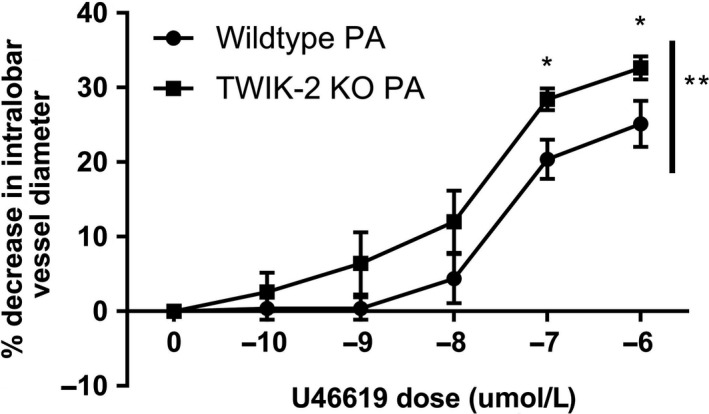
D‐PA from Twik‐2^−/−^ and WT mice in PA conditions(venous) buffer. A U46619 dose titration curve showing altered vasoreactivity in the Twik‐2^−/−^ D‐PA (*P* < 0.05), particularly at the higher concentrations of U46619. *n* = 5 animals per group.

We then measured D‐PA vasocontractile responses to the vasoconstrictor U46619. U46619 is a potent vasoconstrictor like phenylephrine, but differs in its mode of action through its binding thromboxane A_2_ G‐protein receptor. We tested the D‐PA responses a under physiologic (venous) conditions. Figure 6 demonstrates the U46619 titration curve of the Twik‐2^−/−^ D‐PA is hypercontractile compared to the WT D‐PA vessels. Based on the vessel responses to both vasoconstrictors (PE and U46619) the Twik‐2 D‐PA vessel are more hypercontractile based on anatomic region as well as genotype, regardless of the class of vasoconstrictor.

## Discussion

In this series of studies, we demonstrate the following: (1) Twik‐2^−/−^ mice have enhanced vasocontractility in the distal pulmonary vasculature; (2) this hypercontractility is part due increased depolarization in the PASMCs of mice resistance vessels relative to the conduit vessels of the proximal pulmonary vasculature, as well as parallel increased PASMC cytosolic Ca^2+^ concentration in the PASMCs from resistance vessels; (3) application of physiologic venous conditions in pulmonary pressurized vessel studies ensures accurate vasoreactivity measurements that are not revealed in the standard arterial conditions that have been previously reported but do not mimic the pulmonary vascular milieu. These studies expand on our previous studies utilizing a genetic model of *KCNK6 (Twik‐2)* gene deletion. This current set of studies demonstrates a putative cellular mechanism for the development of pulmonary hypertension: that loss of function of Twik‐2 leads to enhanced pulmonary vasoconstriction through increased intracellular calcium levels in the distal PASMCs and depolarization of the distal PASMCs. This hypercontractile response of the distal PASMCs appears irrespective of the class of vasoconstrictor (at least PE and thromboxane A2.) Importantly, these studies demonstrate that hypercontractility varies along the anatomic region of the mouse pulmonary vascular bed, and our novel ability to perfuse and pressurize pulmonary vessels from varying regions with simulated physiologic conditions enhances our ability to study these functional differences based on the anatomic microenvironment.

By isolating a whole mouse pulmonary vessel and applying constant intraluminal pressure to replicate in vivo conditions, these studies provide further insights into the role that Twik‐2 loss‐of‐function plays in pulmonary vasoreactivity. Previously published work in pressurized cerebral and rat vessels (Jernigan et al. 2012; Crossland et al. 2013; Durgan et al. 2015; Ko et al. 2010) did not represent the physiologic venous conditions of the pulmonary resistance vessels in the manner of these current mouse studies were conducted. Thus these studies reflect both the anatomic site and conditions that lead to the vasculopathy of pulmonary hypertension. Our current pressurized and perfused vessel model therefore expands and enhances published techniques in a more physiologically representative manner (Jernigan et al. 2004, 2006, 2012; Broughton et al. 2008, 2010; Fike et al. 2012; Yang et al. 2012; Norton et al. 2013; Artem'eva et al. 2015; Kitagawa et al. 2017) Our previous study on another K_2_P channel,Task‐1 demonstrated that the loss of function of this particular K_2_P channel did not appear to exert any effect on the development of pulmonary hypertension in the same way Twik‐2 dysfunction contributed to pulmonary vasocontractility (Kitagawa et al. 2017).This study builds on our previous work and convincingly shows that Twik‐2 loss‐of‐function exerts its affects preferentially in the distal vasculature. The current set of studies also poses the rationale for future studies in pulmonary hypertension to preferentially utilize the distal pulmonary vasculature. These data in a mouse model of pulmonary hypertension reveal that anatomic heterogeneity confers functional differences across the pulmonary vasculature in terms of electrophysiologic and vasocontractile properties. This variation in responses to vasoconstrictors between different anatomic niches in the mouse pulmonary vasculature suggest that further studies are needed to delineate how K_2_P channels are distributed across the pulmonary vascular anatomy and how these channels are involved in intracellular processes that regulate PASMC contraction across the microvasculature. As many intracellular vasoconstrictor pathways are activated by agonists such as rho‐kinase and endothelin‐1 that signal through G‐protein receptors, it is important to further characterize what is known about the interaction between K_2_P channels and G‐protein receptor signaling (Mathie 2007; Barman et al. 2007). Chronic downregulation or inactivation of the background K_2_P channels (such as Twik‐2) could be a depolarizing trigger leading to enhanced vascular smooth muscle cell G‐protein receptor signaling and pulmonary hypertension. The Twik‐2 channel's role in determining PASMC contractility requires more clarification but these studies suggest that enhancement of Twik‐2 activity could be a potential target of future therapies in treating pulmonary hypertensive disease. These studies reveal the variability between the anatomic regions of the pulmonary vasculature, particularly in that the distal vasculature has enhanced vasoreactivity relative to the proximal portion of the vascular bed, an observation which supports the distal arteriopathy that defines pulmonary hypertension.

## Conflict of Interest

None of the authors disclose any potential sources of conflict of interest.

## References

[phy213950-bib-0001] Antigny, F. , A. Hautefort , J. Meloche , M. Belacel‐Ouari , B. Manoury , C. Rucker‐Martin , et al. 2016 Potassium‐channel subfamily K‐member 3 (KCNK3) contributes to the development of pulmonary arterial hypertension. Circulation 133:1371–1385. 26912814 10.1161/CIRCULATIONAHA.115.020951

[phy213950-bib-0002] Artem'eva, M. M. , Y. O. Kovaleva , O. S. Medvedev , and N. A. Medvedeva . 2015 Effect of chronic administration of estradiol on responsiveness of isolated systemic and pulmonary blood vessels from ovariectomized wistar rats with hypoxic pulmonary hypertension. Bull. Exp. Biol. Med. 159:427–430. 26395625 10.1007/s10517-015-2982-x

[phy213950-bib-0003] Barman, S. A . 2007 Vasoconstrictor effect of endothelin‐1 on hypertensive pulmonary arterial smooth muscle involves Rho‐kinase and protein kinase C. Am. J. Physiol. Lung Cell. Mol. Physiol. 293:L472–L479. 17468135 10.1152/ajplung.00101.2006

[phy213950-bib-0004] Bohnen, M. S. , D. Roman‐Campos , C. Terrenoire , J. Jnani , K. J. Sampson , W. K. Chung , et al. 2017 The impact of heterozygous KCNK3 mutations associated with pulmonary arterial hypertension on channel function and pharmacological recovery. J. Am. Heart Assoc. 6:e006465. 28889099 10.1161/JAHA.117.006465 PMC5634293

[phy213950-bib-0005] Broughton, B. R. S. , B. R. Walker , and T. C. Resta . 2008 Chronic hypoxia induces Rho kinase‐dependent myogenic tone in small pulmonary arteries. Am. J. Physiol. Lung Cell. Mol. Physiol. 294:L797–L806. 18263668 10.1152/ajplung.00253.2007

[phy213950-bib-0006] Broughton, B. R. S. , N. L. Jernigan , C. E. Norton , B. R. Walker , and T. C. Resta . 2010 Chronic hypoxia augments depolarization‐induced Ca2 + sensitization in pulmonary vascular smooth muscle through superoxide‐dependent stimulation of RhoA. Am. J. Physiol. Lung Cell. Mol. Physiol. 298:L232–L242. 19897743 10.1152/ajplung.00276.2009 PMC2822557

[phy213950-bib-0007] Crossland, R. F. , D. J. Durgan , E. E. Lloyd , S. C. Phillips , A. K. Reddy , S. P. Marrelli , et al. 2013 A new rodent model for obstructive sleep apnea: effects on ATP‐mediated dilations in cerebral arteries. Am. J. Physiol. Regul. Integr. Comp. Physiol. 305:R334–R342. 23761641 10.1152/ajpregu.00244.2013 PMC3833398

[phy213950-bib-0008] Durgan, D. J. , R. F. Crossland , E. E. Lloyd , S. C. Phillips , and R. M. Bryan . 2015 Increased cerebrovascular sensitivity to endothelin‐1 in a rat model of obstructive sleep apnea: a role for endothelin receptor B. J. Cereb. Blood Flow Metab. 35:402–411. 25425077 10.1038/jcbfm.2014.214 PMC4348382

[phy213950-bib-0009] Enyedi, P. , and G. Czirjak . 2010 Molecular background of leak K+ currents: two‐pore domain potassium channels. Physiol. Rev. 90:559–605. 20393194 10.1152/physrev.00029.2009

[phy213950-bib-0010] Fike, C. D. , M. Kaplowitz , Y. Zhang , M. Dantuma , and J. A. Madden . 2012 Effect of a phosphodiesterase 5 inhibitor on pulmonary and cerebral arteries of newborn piglets with chronic hypoxia‐induced pulmonary hypertension. Neonatology. 101:28–39. 21791937 10.1159/000326270 PMC3151003

[phy213950-bib-0011] Galiè, N. , M. Humbert , J. L. Vachiery , S. Gibbs , I. Lang , A. Torbicki , et al. 2015 2015 ESC/ERS Guidelines for the diagnosis and treatment of pulmonary hypertension. Eur. Respir. J. 46:1855–1856.26621899

[phy213950-bib-0012] Gardener, M. J. , I. T. Johnson , M. P. Burnham , G. Edwards , A. M. Heagerty , and A. H. Weston . 2004 Functional evidence of a role for two‐pore domain potassium channels in rat mesenteric and pulmonary arteries. Br. J. Pharmacol. 142:192–202. 15066906 10.1038/sj.bjp.0705691 PMC1574915

[phy213950-bib-0013] Jernigan, N. L. , B. R. Walker , and T. C. Resta . 2004 Chronic hypoxia augments protein kinase G‐mediated Ca2 + desensitization in pulmonary vascular smooth muscle through inhibition of RhoA/Rho kinase signaling. Am. J. Physiol. Lung Cell. Mol. Physiol. 287:L1220–L1229. 15310556 10.1152/ajplung.00196.2004

[phy213950-bib-0014] Jernigan, N. L. , B. R. S. Broughton , B. R. Walker , and T. C. Resta . 2006 Impaired NO‐dependent inhibition of store‐ and receptor‐operated calcium entry in pulmonary vascular smooth muscle after chronic hypoxia. Am. J. Physiol. Lung Cell. Mol. Physiol. 290:L517–L525. 16243900 10.1152/ajplung.00308.2004

[phy213950-bib-0015] Jernigan, N. L. , L. M. Herbert , B. R. Walker , and T. C. Resta . 2012 Chronic hypoxia upregulates pulmonary arterial ASIC1: a novel mechanism of enhanced store‐operated Ca2 + entry and receptor‐dependent vasoconstriction. Am. J. Physiol. Cell Physiol. 302:C931–C940. 22205392 10.1152/ajpcell.00332.2011 PMC3311238

[phy213950-bib-0016] Kitagawa, M. G. , J. O. Reynolds , X. H. T. Wehrens , R. M. Jr Bryan , and L. M. Pandit . 2017 Hemodynamic and pathologic characterization of the TASK‐1‐/‐ mouse does not demonstrate pulmonary hypertension. Front Med (Lausanne). 4:177. 29109948 10.3389/fmed.2017.00177 PMC5660113

[phy213950-bib-0017] Ko, E. A. , M. Y. Song , R. Donthamsetty , A. Makino , and J. X. Yuan . 2010 Tension measurement in isolated rat and mouse pulmonary artery. Drug Discovery Today: Disease Models 7:123–130. 23175638 10.1016/j.ddmod.2011.04.001 PMC3501740

[phy213950-bib-0019] Lloyd, E. E. , R. F. Crossland , S. C. Phillips , S. P. Marrelli , A. K. Reddy , G. E. Taffet , et al. 2011 Disruption of K2P6.1 produces vascular dysfunction and hypertension in mice. Hypertension 58:672–678. 21876070 10.1161/HYPERTENSIONAHA.111.175349 PMC3205080

[phy213950-bib-0020] Ma, L. , D. Roman‐Campos , E. D. Austin , M. Eyries , K. S. Sampson , F. Soubrier , et al. 2013 A novel channelopathy in pulmonary arterial hypertension. N. Engl. J. Med. 369:351–361. 23883380 10.1056/NEJMoa1211097 PMC3792227

[phy213950-bib-0021] Mathie, A. 2007 Neuronal two‐pore‐domain potassium channels and their regulation by G protein‐coupled receptors. J. Physiol. 578(Pt 2):377–385. 17068099 10.1113/jphysiol.2006.121582 PMC2075148

[phy213950-bib-0022] Norton, C. E. , B. R. Broughton , N. L. Jernigan , B. R. Walker , and T. C. Resta . 2013 Enhanced depolarization‐induced pulmonary vasoconstriction following chronic hypoxia requires EGFR‐dependent activation of NAD(P)H oxidase 2. Antioxid. Redox Signal. 18:1777–1788. 22966991 10.1089/ars.2012.4836 PMC3619151

[phy213950-bib-0023] Olschewski, A. , Y. Li , B. Tang , J. Hanze , B. Eul , R. M. Bohle , et al. 2006 Impact of TASK‐1 in human pulmonary artery smooth muscle cells. Circ. Res. 98:1072–1080. 16574908 10.1161/01.RES.0000219677.12988.e9

[phy213950-bib-0024] Pandit, L. M. , E. E. Lloyd , J. O. Reynolds , W. S. Lawrence , C. Reynolds , X. H. Wehrens , et al. 2014 TWIK‐2 channel deficiency leads to pulmonary hypertension through a rho‐kinase–mediated process. Hypertension 64:1260–1265. 25245387 10.1161/HYPERTENSIONAHA.114.03406 PMC4231005

[phy213950-bib-0025] Patel, A. J. , F. Maingret , V. Magnone , M. Fosset , M. Lazdunski , and E. Honoré . 2000 TWIK‐2, an inactivating 2P domain K+ channel. J. Biol. Chem. 275:28722–28730. 10887187 10.1074/jbc.M003755200

[phy213950-bib-0026] Ryan, J. J. , and S. L. Archer . 2015 Recent advances in pulmonary hypertension: emerging concepts in the molecular basis of pulmonary arterial hypertension. Circulation 131:1691–1702. 25964279 10.1161/CIRCULATIONAHA.114.006979 PMC4429908

[phy213950-bib-0027] Salinas, M. , R. Reyes , F. Lesage , M. Fosset , C. Heurteaux , G. Romey , et al. 1999 Cloning of a new mouse two‐P domain channel subunit and a human homologue with a unique pore structure. J. Biol. Chem. 274:11751–11760. 10206991 10.1074/jbc.274.17.11751

[phy213950-bib-0028] Schermuly, R. T. , H. A. Ghofrani , M. R. Wilkins , and F. Grimminger . 2011 Mechanisms of disease: pulmonary arterial hypertension. Nat. Rev. Cardiol. 8:443–455. 21691314 10.1038/nrcardio.2011.87 PMC7097518

[phy213950-bib-0029] Sutendra, G. , and E. D. Michelakis . 2013 Pulmonary arterial hypertension: challenges in translational research and a vision for change. Sci. Transl. Med. 5:208sr5. 24154604 10.1126/scitranslmed.3005428

[phy213950-bib-0031] Yang, X. R. , A. H. Lin , J. M. Hughes , N. A. Flavahan , Y. N. Cao , W. Liedtke , et al. 2012 Upregulation of osmo‐mechanosensitive TRPV4 channel facilitates chronic hypoxia‐induced myogenic tone and pulmonary hypertension. Am. J. Physiol. Lung Cell. Mol. Physiol. 302:L555–L568. 22207590 10.1152/ajplung.00005.2011 PMC3311528

